# Influences on birth spacing intentions and desired interventions among women who have experienced a poor obstetric outcome in Lilongwe Malawi: a qualitative study

**DOI:** 10.1186/s12884-018-1835-9

**Published:** 2018-05-31

**Authors:** Dawn M. Kopp, Agatha Bula, Suzanne Maman, Lameck Chinula, Mercy Tsidya, Mwawi Mwale, Jennifer H. Tang

**Affiliations:** 1 UNC Project-Malawi, Private Bag, A-104 Lilongwe, Malawi; 2 UNC Department of Obstetrics & Gynecology, Chapel Hill, NC USA; 3 Kamuzu Central Hospital, Lilongwe, Malawi; 4 UNC Department of Health Behavior, Chapel Hill, NC USA; 5 Malawi College of Medicine Department of Obstetrics & Gynaecology, Blantyre, Malawi; 6 Bwaila Hospital, Lilongwe District Health Office, Lilongwe, Malawi

**Keywords:** Birth spacing, Stillbirth, Neonatal death, Malawi, Africa

## Abstract

**Background:**

Stillbirth and neonatal mortality are very high in many low-income countries, including Malawi. Use of family planning to encourage birth spacing may optimize outcomes for subsequent pregnancies. However, reproductive desires and influences on birth spacing preferences of women who have experienced a stillbirth or neonatal death in low-resource settings are not well understood.

**Methods:**

We conducted a qualitative study using 20 in-depth interviews and four focus group discussions with women who had experienced a stillborn baby or early neonatal death to explore attitudes surrounding birth spacing and potential interventions to promote family planning in this population. Qualitative data were analyzed for recurrent patterns and themes and central ideas were extracted to identify their core meanings.

**Results:**

Forty-six women participated in the study. After experiencing a stillbirth or neonatal death, most women wanted to wait to become pregnant again but women with living children wished to wait for longer periods of time than those with no living children. Most women preferred birth spacing interventions led by clinical providers and inclusion of their spouses.

**Conclusions:**

Many influences on family size and birth spacing were noted in this population, with the most significant influencing factor being the spouse. Interventions to promote birth spacing and improve maternal and neonatal health in this population need to involve male partners and knowledgeable health care providers to be effective.

**Trial registration:**

Clinicaltrials.gov NCT02674542 Registered February 1, 2016 (retrospectively registered).

**Electronic supplementary material:**

The online version of this article (10.1186/s12884-018-1835-9) contains supplementary material, which is available to authorized users.

## Background

Stillbirths and neonatal deaths remain high in many low-income countries. Worldwide, there are an estimated 2.64 million stillbirths and 2.0 million early neonatal deaths yearly [1]. The perinatal death rate in Malawi is 35 per 1000 pregnancies of 7 or more months gestation [2], much higher than a rate of 6.51 in high income countries such as the United States. [3]. The leading cause of neonatal death in Malawi is preterm birth [4]. Several retrospective studies have shown an association between a short interpregnancy interval (IPI) and adverse maternal and newborn outcomes: low birth weight (LBW), small for gestational age (SGA), preterm birth, premature preterm rupture of membranes (PPROM), and maternal death [5]. Adverse perinatal outcomes are seen with IPI less than 18 months [6–8]. In Malawi, pregnancies that occurred fewer than 15 months after the previous pregnancy have the highest perinatal mortality rate (55 deaths per 1000 pregnancies) when compared to other birth spacing intervals [2]. The World Health Organization (WHO) currently recommends an IPI of at least 24 months to reduce infant and child mortality as well as to benefit maternal health [9].

Few Malawian women have a birth interval less than 18 months (2.9%) if the preceding child is living at the time of the next birth. However, this proportion increases to 22.8% if the preceding birth’s status is deceased at the time of the next birth [2]. It is unclear if women in this situation desire short IPIs, are influenced to do so by people or situations, or are aware of the risks associated with short IPI.

There may be a role for postpartum birth spacing education and provision of family planning to increase IPIs in this population. Postpartum contraception has been shown to be protective against preterm birth [10]. Though lactational amenorrhea is a very effective method of contraception in the first 6 months postpartum [11] and breastfeeding rates in this time period are very high in Malawian and other sub-Saharan African populations [12, 13], women with a stillbirth or neonatal death are unable to use lactational amenorrhea as a family planning method as they have no living child to breastfeed. In low-resource settings, the rates and reasons for postpartum contraceptive use for birth spacing after a neonatal death or stillbirth are largely unknown.

Understanding the attitudes surrounding future fertility and birth spacing in this population is critical to propose socially and culturally acceptable interventions to address their potential unmet need for family planning. Therefore, we conducted a qualitative study to obtain data exploring birth spacing intentions, influences on these intentions, and preferred modes of birth spacing interventions among Malawian women who had experienced poor obstetric outcomes.

## Methods

This was a qualitative study using in-depth, semi-structured interviews and focus group discussions. Approval was obtained from the National Health Sciences Research Committee of Malawi (Protocol #1354) and the University of North Carolina School of Medicine Institutional Review Board (#14–2677). Women gave written informed consent at the time of enrollment in the language of their choice (Chichewa or English).

The study population was recruited from Bwaila Hospital, a district government hospital in Lilongwe, the capital city of Malawi. Bwaila has approximately 15,000 deliveries annually, of which 2900 are preterm. Between 80 and 110 cases of birth asphyxia (a portion of which results in stillbirth or neonatal death) occur each month on the labor ward. Inclusion criteria for the study were: 1) current admission to the postpartum ward at Bwaila Hospital; 2) delivery of a stillborn over 28 weeks gestation or with a birthweight ≥1000 g, or delivery of a liveborn infant weighing ≥1000 g with a neonatal demise in the first 7 days of life; 3) ability to speak Chichewa (the local language) or English fluently; and 4) age 18–45 years old.

We recruited 60 women with and without living children from prior pregnancies at a 1:1 ratio from the postnatal wards. A demographic form was completed for women who consented to be part of the study. This form collected information about age, number of other living children, HIV status, marital status, completed education, and occupation. Data on access to a working phone and roof type were collected to assess socioeconomic status. HIV testing is performed on all Malawian women during antenatal care unless they opt out. HIV status was determined by verifying the participant’s health passport (a government-issued personal medical record booklet kept by the patient) with the participant’s permission at time of enrollment.

Enrolled women were then contacted and traced 4–8 weeks later to either participate in an in-depth interview or a focus group discussion. All in-depth interviews and focus group discussions were conducted by the same experienced bilingual researcher (M.T.). Twenty in-depth interviews took place in participants’ homes or another private setting and four focus group discussions (of 6–8 participants each) took place in a private conference room on the campus of Kamuzu Central Hospital in Lilongwe, Malawi. Interviews and focus group discussions were audiotaped, transcribed and translated into English. All transcriptions and translations were completed by the same researcher (M.T.). Accuracy of the translations was verified by two other bilingual members of the research team (A.B. and G.H.).

Our analysis approach was to use content analysis to compare the birth spacing intentions of women who did and did not have living children. The interview and focus group discussion guide (Additional files 1 and 2) focused on several domains, two of which are relevant to this analysis: 1) birth spacing plans and influences, and 2) acceptable educational interventions to promote birth spacing and family planning among women with poor obstetric outcome. Focus group and in-depth interview guides were used to ensure that all critical topics were discussed, but the interviewer was given license to cover topics in a manner that facilitated flow and rapport. A specific aim of the focus group discussions was to facilitate brainstorming about potential birth spacing interventions, whereas the in-depth interviews focused more on individual and social influences on birth spacing that may be too personal to share in a group setting. For each domain, results were analyzed separately for women with and without living children prior to the stillbirth or neonatal death to examine the role this plays on reproductive desires.

Previous qualitative exploration in this field has demonstrated that the minimum threshold for data saturation can be reached within 20 in-depth interviews and four focus group discussions [14–16]. Transcripts of completed interviews were independently analyzed by two of the investigators (A.B. and D.K.). A code dictionary was developed in an iterative process based on identified domains, and this dictionary was assigned to sections of the text using qualitative software NVivo® 10. Recurrent themes and sub-themes were identified based on these initial codes, and any discrepancies were resolved through discussion. Matrices and tables were used to organize the data and display these to facilitate analysis that integrated both in-depth interviews and focus group discussions based on the conceptual domains determined a priori.

## Results

### Participant characteristics

Of the 60 women enrolled in the study, 46 women participated in focus group discussions or in-depth interviews 4–8 weeks after delivery. Twenty in-depth interviews and four focus groups with 6–7 women per group were conducted. Although 30 women with living children and 30 women without living children were recruited, slightly more women who participated in the study (*n* = 28, 61%) had living children (Table 1). Most women were between the ages of 18–34 years, and women without living children were younger than those with living children. Seven women (15%) were HIV-infected. Those with living children had a median of 2 children (range 1–8). More women with living children were married, HIV-infected, had no education, and had a metal, wood, or cement roof. During the interviews and focus groups, many sub-themes emerged under content analysis (Table 2), which revealed the dynamics behind reproductive decision-making in this group of women.

**Table 1:** Participant Characteristics *n* = 46 (20 in-depth interview participants and 26 focus group discussion participants)

Characteristic	All participants (*n* = 46) n (%)	Women with living children (*n* = 28) n (%)	Women without living children (*n* = 18) n (%)
Age			
18–24 years	24 (52)	8 (29)	16 (89)
25–34 years	16 (35)	14 (50)	2 (11)
≥35 years	6 (13)	6 (21)	0 (0)
Pregnancy Outcome			
Stillbirth	23 (50)	14 (50)	9 (50)
Neonatal death	23 (50)	14 (50)	9 (50)
Marital Status			
Married	39 (85)	28 (100)	11 (61)
Not married	7 (15)	0 (0)	7 (39)
HIV status			
HIV-uninfected	39 (85)	22 (79)	17 (94)
HIV-infected	7 (15)	6 (21)	1 (6)
Religion			
Christian	37 (80)	22 (79)	15 (83)
Muslim	9 (20)	6 (21)	3 (17)
Education			
None	12 (26)	9 (32)	3 (17)
Some primary	21 (46)	13 (46)	8 (44)
Secondary or more	13 (28)	6 (21)	7 (39)
Phone			
Has working phone	17 (37)	12 (43)	5 (28)
No working phone	29 (63)	16 (57)	13 (72)
Roof type			
None/Grass	16 (35)	8 (29)	8 (44)
Metal/wood/cement	29 (63)	20 (71)	9 (50)

**Table 2:** Domains, categories, themes, and sub-themes from interviews and focus groups with Malawian women after a poor obstetric outcome

Domain	Theme	Sub-theme
Birth spacing plans and influences	a. Biological	1) Role of the incident pregnancy and number of living children
		2) Return to fertility: correct and incorrect knowledge
		3) Gaining strength after birth
		4) Influence of delivery experience/maternal health
		5) Replacing the deceased child
	b. Social	1) Care for existing children/preparing for next child
		2) Husband’s desires/Concerns about marital conflict
		3) Influence of family/friends
Acceptable educational interventions to promote birth spacing	a. Personal experience with birth spacing education	
	b. Recommendations for birth spacing interventions	1) Timing of birth spacing intervention
		2) Location of birth spacing intervention
		3) Providers of birth spacing intervention
		4) Group or individual sessions
		5) Involving men in birth spacing intervention

## Birth spacing plans and influences

We identified two themes of influence on birth spacing plans among women who had experienced poor obstetric outcome: biological and social. These two themes were then broken down into relevant sub-themes.

### Biological

#### Role of the incident pregnancy and number of living children

All but one woman agreed that the birth spacing interval should be shorter for women who had experienced a stillbirth or neonatal death than those who had experienced a live birth of a healthy newborn, though they did not agree on exactly how long this waiting time should be. The one woman who felt birth spacing intervals would be the same had no living children and was not married.

*“Because if the child is alive, it is understood and even the husband can agree that you should use child spacing methods, but if the child is not alive, there is no way you can wait for a longer time…But not that the child spacing period between the child that is dead can be the same as that of the child that is alive because you can have a spacing of three or four years for a child that is alive and this cannot apply to the child that is dead. The woman with a child that is dead can only wait for some months or one year.”* (Focus group 1, participant no. 4, age 18–24 years, no living children).

All women were also asked about their personal plans for birth spacing. There were differences between those who had living children and those who did not. Among the 28 women with living children, four did not want any additional children and were planning to obtain sterilization. None of the women without living children expressed a plan for sterilization.

Of those who desired more children, most women wanted to wait to become pregnant again. The amount of time women wished to wait until their next pregnancy varied by whether or not they had living children. Most women with living children wished to wait at least 2 years, whereas most of those without living children wished to wait 1–12 months before attempting pregnancy.

#### Return to fertility: Correct and incorrect knowledge

Most women correctly named having sexual intercourse before initiating family planning methods as being a main influence on birth spacing. Several women recognized that they and other women who just had a stillbirth or early neonatal death might have a higher fertility after delivery than those who recently gave birth to a living child.

*“There is a difference between a woman who has a live child and the one who has a child that did not live. The one with a live child can stay for one year without pregnancy because she is breastfeeding but for someone who has a child that did not live she can become pregnant soon [after] she resumes sex.”* (In-depth interview participant no. 13, age 25–34 years, 6 living children).

However, not all women understood the signs and symptoms of a return to fertility postpartum. Specifically, some thought that women could not get pregnant if they still had some bleeding after childbirth (lochia). Other women incorrectly noted that before normal menstrual cycles resume again, women are unable to become pregnant.

#### Gaining strength after birth

Nearly all women cited the need to gain strength and/or energy after birth as an influencing factor on birth spacing. Most women also closely associated gaining of strength with the replacement of blood lost during delivery.

Interviewer: “*Why do you want to become pregnant again after two years?”*Participant: “*My body should regain its strength and it should be strong enough to accommodate another pregnancy. Because it happens that during labor and delivery, a woman may lose a lot of blood. So the blood which was lost during delivery has to be replaced before she gives birth to another child. By the end of two years going upwards, the woman has regained her energy and the blood that she lost has been replaced.”* (In-depth interview participant no. 7, age 25–34 years, 1 living child).

#### Influence of delivery experience/maternal health

Women also cited their prior delivery experience as a factor influencing them to wait before getting pregnant again. However, the way this experience influenced them differed. Some women said that mode of delivery influenced birth spacing.

Interviewer: “*Why after two years?”*

Participant*: “Because of the problem of cesarean…because the wound by then is not yet healed.”*

Interviewer: “*What if she had normal delivery?”*Participant*: “She can wait for one year.”* (In-depth interview participant no. 3, age 18–24 years, 1 living child).

Other women pointed out that they had experienced medical complications that caused them to desire longer waiting periods before achieving another pregnancy. These women pointed out that perhaps by waiting longer, they might be able to avoid these complications or more severe exacerbations of these conditions in future pregnancies.

Interviewer*: “What factors would encourage women to use family planning after having babies that are living?”*

Focus group 1 participant no. 4: *“The problems that the women went through during labor and delivery.”*

Interviewer: “*Like what problems?”*

Focus group 1 participant no. 4: *“Say during pregnancy the woman was experiencing high blood pressure, anemia, she can be afraid to become pregnant soon. She can decide to wait…so while waiting for the time to become pregnant again the women will need to use reliable family planning methods.”* (age 18–24 years, no living children).

A few women specifically felt that their health condition made any future pregnancies risky and influenced their decision to seek permanent sterilization to have no more future pregnancies.

Interviewer: “*Why do you want to go for permanent contraception?”*

Participant*: “I am HIV positive, and it just happened that I became pregnant because my new husband wanted a child, but I had many complications during pregnancy, labor and delivery, and had it been that I was not rushed to Bwaila [Hospital] I would have lost my life. So I don’t want to become pregnant again.”* (In-depth interview participant no. 17, age ≥35 years, 4 living children).

Though 15% of participants were HIV-infected, this was the only mention of HIV status as an influence on birth spacing. No differences in frequency or type of responses were seen on the influences of birth spacing between HIV-infected and HIV-uninfected women.

More than half of the women seemed to have almost no understanding about associated conditions or complications that led to their child’s stillbirth or neonatal death. For these women unanswered questions about how they could prevent this in future pregnancies influenced them to wait before attempting another pregnancy. Another way delivery experience influenced women was through psychological trauma. Some women noted that dealing with loss of a child they had been anticipating for months would be alive was difficult, and they would need time to emotionally recover from this.

*“Sometimes you are afraid after experiencing a stillbirth because you never know what went wrong in your womb and this can encourage you to be on family planning method before becoming pregnant again. Also fear. Like myself, I am always afraid of experiencing the same problem. So I feel like it is better to take family planning methods so that may be time can help me to recover from the trauma that I went through.”* (Focus group 1, participant no. 7, age 18–24 years, no living children).

#### Replacing the deceased child

Nearly all women cited an impulse to replace the child that was recently lost with another pregnancy as a strong influencer on birth spacing after poor obstetric outcome. Few women expressed that this was an internal motivating factor, but many more expressed that the husband was pushing this perspective within the family.

Interviewer: “*Why do you think the woman should wait for one year if she gave birth to a child that did not live”?*

Participant: “*Because the husband may say that he wants a child in the house. So the family has to forge ahead and not just to be disappointed because they had a child that did not live”.* (Focus group 3, participant no.2, age 18–24 years, 1 living child).

A related concept to the need to replace the child that had passed was termed by several women both in in-depth interviews and in focus groups as “spacing the grave”.

Participant: *“Because here in the village, people talk a lot. ‘You are using injection to space the grave?’”*Interviewer: “*What does ‘spacing the grave mean?”*Participant: “*Meaning that you are using child spacing methods for the child who died.”* (In-depth interview participant no. 3, age 18–24 years, 1 living child).

*“But if the child did not live the time will be short: maybe six months later the woman should become pregnant again because it is said that you cannot space a child that you don’t see.”* (In-depth interview participant no. 8, age ≥35 years, 8 living children).

It seems that these women had interpreted or had others around them interpret the concept of birth spacing as delaying the interval between one *live* birth and the next pregnancy. To women using this definition, birth spacing cannot and does not apply to women who don’t have a living child.

### Social

#### Caring for existing children/preparing for the next child

When discussing a need to space their pregnancies, some women felt birth spacing time should allow them to be able to care for their existing children or prepare for their next child. They did not make a distinction between their experience of having had stillbirth or neonatal death and other women whose pregnancy resulted in a live child. Other women noted that birth spacing was important for financial reasons, such as being able to spend more time farming or growing their business to have the resources to buy and provide the necessities for raising future children.

*“…we want to work in our farm and see how best we can take care of the children that we already have. So even we already had plans of waiting to take care of our children before I become pregnant again.”* (In-depth interview participant no. 9, age 18–24 years, 3 living children).2

#### Husband’s desires/concerns about marital conflict

Although several women stated that their husband agreed with their plans for birth spacing, many discussed a conflict or a potential conflict between their views and the views of their spouses. In all cases, the conflict arose because women wanted to wait longer to become pregnant than the husband wanted to wait.

Interviewer: “*How about your husband, when would he want you to be pregnant again?”*

Participant: *“He may want me to be pregnant very soon but that may not be a good idea.”*

Interviewer: “*What reason may your husband have for wanting you to be pregnant very soon?”*

Participant*: “It’s because you know most men do not understand the suffering that a woman goes through during pregnancy and childbirth.”*

Interviewer: “*Now according to what happened to you during your last child’s birth, what reason might your husband have for wanting you to be pregnant soon?”*

Participant: “*According to what happened, he might think that the solution is getting another child soon.”* (In-depth interview participant no. 19, age 18–24 years, 3 living children).

Women also noted that it would be a disadvantage to women to wait 18 months or more after stillbirth or neonatal death to become pregnant again because it will cause marital conflict. Many women discussed that husbands routinely make decisions regarding family planning use and timing of pregnancy.

*“…normally men are the ones who facilitate decisions of not using family planning methods. Sometimes a woman may think of taking family planning methods, but if the husband says ‘no’, the woman has no say and to avoid quarrels in the house that woman will just follow what the husband has said.”* (Focus group 1, participant no. 4, age 18–24 years, no living children).

One woman stated that a conflict over pregnancy timing might lead to dissolution of marriage:

Interviewer: “*What may bring the misunderstandings?”*

Participant: “*It may be that she may want to use [a] family planning method while her husband wants another child; so it may bring chaos in the family with the man telling the woman that if she goes for contraceptives her marriage will be over. The woman may choose not to go for the methods.”* (In-depth interview participant no. 10, age 18–24 years, 2 living children).

Four women noted that this marital conflict could lead to the husband to have affairs to have children with other women.

Interviewer: *“What problems could happen to the mother for getting pregnant more than 18 months after giving birth to a baby that did not live?”*

Participant: “*The problem could be there if the man wanted to have a baby before the waiting period of his wife [is completed], he can go outside to have other women. In so doing he can be infected and later infect the mother and that can have an impact if the mother was to have a child after the waiting period.”* (Focus group 1, participant no. 4, age 18–24 years, no living children).

Another woman felt that this marital conflict might be dangerous for the woman because if a wife does not want to have a child soon after one that died, it could implicate her for playing a role in the child’s death.

*“The problem that can be there is conflict in the family. The husband may think that the woman is deliberately not becoming pregnant because she doesn’t want to have a child, and he may also think that the wife killed the child deliberately so that she should not have a child.”* (Focus group 3, participant no. 6, age 30 years, 2 living children).

#### Influence of family/friends

Unlike the influence of husbands, women reported that their family and friends were more divided: either agreeing with their preferred birth spacing or advising either more or less time before becoming pregnant again. Family and friends seemed to often express concern over the woman’s health and remind women of the difficult and stressful delivery they had just experienced.

*“I can also add that it can take the challenges that the woman went through during labor and delivery because most of the times they are the friends and relatives who know the problems that the woman went through during labor and delivery. This is when the friends and relatives can have a say as when can be the best time for the woman to become pregnant again.”* (Focus group 1, participant no. 5, age 18–24 years, no living children).

Still other women felt that it is not the role of anyone outside marriage to give advice or have a say in the timing of a couples’ pregnancy.

*“They cannot tell me when I should become pregnant again. It is a family and confidential decision.”* (Focus group 4, participant no. 3, age 25–34 years, 2 living children).

Other women said that they experienced or feared social stigma after undergoing stillbirth or neonatal death from friends and community members.

*“Some people may be talking a lot when they see that you have had a stillbirth. Like other women may say ‘We were all pregnant but look at that one she doesn’t have a baby.’ So due to fear of being insulted, the woman may tell her husband that it is better for them to have a replacement of the child that died.”* (Focus group 3, participant no.6, age 25–34 years, 2 living children).

*“…some people may laugh at her because she doesn’t have a child and she may want to have another child.”* (In-depth interview participant no. 20, age 25–34 years, 3 living children).

## Acceptable educational interventions to promote birth spacing

### Personal experience with birth spacing education

When asked about family planning or birth spacing advice women had received after their stillbirth or neonatal death, only one woman could recall any health provider discussing this either prior to discharge from hospital or at any follow-up visits they had attended since delivery. Perhaps as a result, some women expressed confusion about starting family planning after delivery.

Interviewer: “*For those of you who are not using any family planning method, why are you not using any family planning method?”*

Focus group 2 participant no. 1*: “Like myself, I planned to go to the hospital but I was not told when I can start using a family planning method.”* (age 25–34 years, 3 living children).

For other women, this lack of information exacerbated conflicts between them and their spouses when they were not in agreement about birth spacing plans. One woman describes deliberately misrepresenting the advice given about birth spacing during a discussion with her husband.

Participant: “*I told him that I was told at the hospital to wait for one year so he just agreed.”*

Interviewer: “*Were you told by anybody at the hospital that you should wait for one year?”*

Participant: “*No, but my mother told me that I should not rush to become pregnant again because I need to regain my strength, so one year would be ideal time. I mentioned the doctors because I knew that if I could say it was my mother he would have not accepted it.”* (In-depth interview participant no. 14, age 18–24 years, no living children).

### Recommendations for birth spacing interventions

Next, we asked women about their thoughts about recommendations for birth spacing interventions.

#### Timing of birth spacing intervention

When women were asked when counseling about birth spacing and family planning should be given after experiencing stillbirth or neonatal death, they were divided. Just over half suggested that it should be between 4 and 8 weeks after delivery, before couples resume sexual intercourse. The other women suggested it should take place soon after delivery or at the time the child has died. Some also mentioned that this should take place in the presence of the husband if possible.

Interviewer: “*When would be the best time for women to discuss family planning after having a baby that isn’t living?”*

Focus group 1 participant no. 4: *“Soon after experiencing the stillbirth the woman should start discussing about family planning so that when the family will decide to start having sex, the woman should implement her decision by going for family planning before they start having sex. It is unlike when the issue is discussed the time the family wants to have sex, the woman will be taken for surprise. But if this was done prior, the woman will have a say to the husband that before they start having sex [and] she should go for family planning.”* (age 18–24 years, no living children).

#### Location of birth spacing intervention

Nearly all women agreed that a family planning discussion either in a clinical setting (hospital ward or outpatient clinic) would be acceptable to them. However, one woman mentioned that it would be preferable if the discussion happened at home between the husband and wife prior to a woman going to a clinic and receiving a family planning method.

Few women did not think that they would be able to have an informed discussion about family planning at home because they would not have access to knowledgeable health providers. Though most women felt that health surveillance assistants (local community health workers) would be able to provide adequate contraceptive information to women and couples, others did not feel these providers were appropriately informed or trained.

Women were divided about the necessity of returning to the place of delivery to receive family planning education and contraceptives. Most women felt it was important to go back to the same place because the health providers may remember her delivery complications and be able to ensure she was healthy and to assist her if she was still having issues. However, few women felt that the nearest health facility may be able to provide the same care and counseling. One woman was concerned that returning to the same location may be emotionally painful:

*“Sometimes the care that you received during labor and delivery matters most. Sometimes if the reception at the hospital was poor, you cannot have the desire to go back to that clinic. You would opt to go to a different clinic where you would see different faces.”* (Focus group 1, participant no. 7, age 18–24 years, no living children).

Most women did not feel it was helpful to have a special postnatal clinic for evaluation and counseling of women who had experienced stillbirth or neonatal death. These women feared possible discrimination and stigma. However, other women felt this may be useful for those with chronic conditions that may have led to the poor obstetric outcome. Some thought evaluation and treatment of underlying medical conditions and specialized counseling in such a setting could lead to improved future pregnancy outcomes.

#### Providers of birth spacing intervention

Most women thought that health care providers (health surveillance assistants, nurses, and physicians) would be the most appropriate people to provide birth spacing interventions. Few women felt that fellow women (friends, sisters, mothers, and elderly women in the community) could also lead discussions on family planning.

#### Group or individual sessions for birth spacing intervention

Most women felt that group sessions with groups of women who had all experienced poor obstetric outcome would be beneficial.

Interviewer: “*Do you think it would be useful for women who had children that did not live could be meeting in groups as we have done here at the clinic?”*

Focus group 3 participant no. 3*: “It is useful.”* (age 18–24 years, 2 living children).

Interviewer: “*How?”*

Focus group 3 participant no. 6*: “Because when we experience stillbirths or neonatal deaths, we have worries but when we meet in a group like this one, we feel encouraged that ‘I am not the only person, other women had the similar experience.’ So we share ideas and encourage each other but when you are alone you are stressed up with a lot of worries.”* (age 25–34 years, 2 living children).

However, those women who participated in in-depth interviews expressed concern that this could lead to discrimination and stigma. Some women felt individual sessions to discuss their situation with a health care worker would provide more confidentiality.

#### Involving men in birth spacing interventions

Though many women expressed fear of conflict and feelings of powerlessness in disagreements with their husbands over birth spacing, others thought they could be influenced with education or through being assertive in reproductive conversations. They proposed inviting them to attend and participate in individual or group sessions either in clinical or home-based settings. Women felt that if a couple heard the same health recommendations, they could agree on an appropriate birth spacing interval and family planning method together, which could be beneficial for marriage as well as health of the woman.

Interviewer: “*Do you think it is important to involve men so that they can be providing support to their spouses on issues of family planning?”*

Focus group 4, participant no 3: *“It is important because you can have the same information and there can be trust on each other if each one of you is involved.”* (age 25–34 years, 2 living children).

Focus group 4, participant no 2: *“It can be good if the men can be asked to accompany their spouses to the clinic so that they can be educated as a couple on different family planning methods and the couple should be able to choose the method.”* (age 25–34 years, 3 living children).

Some women advocated standing up for their own health and reproductive desires in their marriage.

*“I just want to add that we as women we don’t need to keep quiet when it comes to childbearing issues. We don’t need to just listen and implement what men tell us. We need to rise and tell the men the truth about our experiences, feelings and opinions regarding childbirth. He should understand that we are the ones who suffer childbirth and we have the right to tell him how long we want to wait before having the next child.”* (Focus group 1, participant no. 5, age 18–24 years, no living children).

## Discussion

These qualitative interviews and focus group discussions revealed the reproductive desires and challenges women face in birth spacing after experiencing poor obstetric outcome. This study shows that the concept of birth spacing is not always thought to apply to those who have experienced a stillbirth or neonatal death. Many women expressed a plan to wait to become pregnant again, with women with no living children wanting to wait less time than those with living children. Husbands were named as strong influencers of family size, family planning use, and birth spacing plans in families. Many women feared marital conflict if they disagreed with their husband’s desire to try again for another pregnancy sooner than they themselves desired, illustrating gender imbalance in reproductive decision-making among couples. Most women felt that involving their husband in birth spacing educational interventions would be beneficial and would help them to understand the need to wait for longer periods between pregnancies, even with poor obstetric outcomes.

Few studies have examined the relationship between poor obstetric outcomes and future pregnancy intentions. A study of HIV-infected adolescents in Kenya did not find an association between poor birth outcomes and postpartum contraceptive use [17]. In a study of postpartum women with a living child in central Malawi, 90% of the women in the study used contraception by 3 months after delivery [18], and in another study of women in northern Malawi 28% used a modern form of contraception by 6 months after delivery [19]. However, neither of these studies examined women whose prior delivery was a stillbirth or neonatal death. Uptake of family planning is affected by attitudes regarding contraceptive methods, which have been shown to be influenced by the perceived and actual adverse effects among Malawians [20]. Qualitative studies involving women who have experienced stillbirth or neonatal death have not previously focused on future pregnancy intentions. Instead, prior studies discuss the invisibility of perinatal loss [21], ways to improve mental health of women [22], or perceptions of care at the time of delivery [23].

In this study, we were able to identify different birth spacing intentions between women with and without living children. Though women were enrolled at a 1:1 ratio for this characteristic, more women with living children could be traced and agreed to participate 4–8 weeks after delivery. However, data from women without living children reached saturation more quickly than from women with living children as the group with living children had a greater variety of perspectives.

The proportion of participants who are HIV-infected was 15%, very similar to the proportion of women who receive antenatal care at Bwaila Hospital (14.7%) [24]. Though this study was conducted in an area with a high background of HIV infection, HIV status was only mentioned once as an influence on birth spacing and no meaningful differences in responses were seen when analyzed by HIV status. Further studies are needed to understand if having a stillbirth or neonatal death impacts postpartum contraceptive use, continuation of antiretroviral therapy, and integration into the health system for HIV-infected women.

There are currently no targeted interventions in Malawi encouraging appropriate birth spacing among women who have experienced poor obstetric outcome. The March of Dimes has instituted a “Wait One Year” program in parts of the United States to encourage women who have experienced preterm birth to use effective contraception to space their pregnancies and decrease the risk of recurrent preterm birth [25]. This program utilizes an educational intervention with a nurse and physician within the first 6 months after preterm delivery and focuses on preterm prevention points, such as smoking cessation, stress reduction, folate supplementation, and dental hygiene, with an emphasis on delaying conception through the use of family planning by at least 12 months after delivery. Similar programs may be effective in a low-income setting where preterm birth, stillbirth, and neonatal death occur at higher rates.

Because of gender inequities and imbalanced power dynamics with reproductive decision-making in marital relationships that was expressed by many participants, we suggest that involvement of men is critical to any intervention to promote healthy birth spacing in this population. Qualitative studies examining couples after stillbirth have found this experience is deeply shared between a woman and her partner, sometimes in a way that isolates them from the community [26]. This experience has also been shown to either have a positive or negative impact on the couples’ relationship [27]. Understanding the complex nature of relationships between men and women who have experienced perinatal loss in an African setting is imperative to effectively encouraging birth spacing in this population.

The discrepant finding between husbands encouraging women to get pregnant soon and family and friends encouraging women to wait may be due to differential involvement in pregnancy and delivery. In Malawi, it is common to have a female guardian, usually a relative or close friend, present with the woman during labor, who is responsible for knowing the health status of the mother and for providing support [28]. Since guardians witness first-hand the pain and complications women have gone through, they are more likely to remind women of this than their husbands, who are not usually allowed in the labor rooms for privacy reasons since the rooms often house more than one laboring woman.

Though not all women agreed on the exact way a birth spacing intervention could be effective for women with perinatal loss, many felt that counseling given by health providers was important. However, only one woman in this study recalled receiving any information on birth spacing after a loss. This may be secondary to avoidance or minimal interaction with these women by health care providers since they do not have living children [23] or low levels of knowledge among providers regarding recommendations for birth spacing after poor obstetric outcome. A survey of American obstetricians found that two-thirds recommended attempting pregnancy less than 6 months after perinatal loss despite literature on the risks of short IPIs to future pregnancies [29]. Assessing knowledge and attitudes of Malawian clinical providers on birth spacing after perinatal loss is important prior to undertaking an intervention that involves their counseling to women and their partners.

## Conclusions

Most women wanted to wait before becoming pregnant again after experiencing stillbirth or neonatal death, but some women felt that birth spacing was not an applicable concept after this outcome. Few women who already had living children wanted no further pregnancies and even desired permanent sterilization, whereas women with no living children were more likely to desire another pregnancy within 1 year. Many influences on family size and birth spacing were noted in this population, with the most significant influencing factor being the spouse and fear of marital conflict. Interventions to promote birth spacing and improve maternal and neonatal health in this population need to involve male partners and knowledgeable health care providers to be effective.

## Additional files

Additional file 1: Guide for focus group discussions. (DOCX 103 kb)

Additional file 2: Guide for in-depth interviews. (DOCX 89 kb)

## Abbreviations

IPI: Interpregnancy interval
No: Number
