# Supplementary material for: Influences on birth spacing intentions and desired interventions among women who have experienced a poor obstetric outcome in Lilongwe Malawi: a qualitative study
Source: BMC Pregnancy Childbirth. 2018 May 31;18:197. doi: 10.1186/s12884-018-1835-9 (PMC5984328; doi:10.1186/s12884-018-1835-9)
Supplement: Supplementary file 2 — Guide for in-depth interviews. (DOCX 89 kb) [file 12884_2018_1835_MOESM2_ESM.docx]

QUESTION GUIDE

**IN-DEPTH INDIVIDUAL INTERVIEWS WITH WOMEN WHO HAVE HAD A STILLBIRTH OR EARLY NEONATAL DEATH**

**Study ID _________________**

Start time ……………. Date…………………………

Place of interview………………………………………………………………….

Future family planning intentions and beliefs

Personal Questions about Family Size

1. How many living children do you have now?
2. Do you want to have any more children? Why?
3. What is the total number of children you want to have? Why?
4. How many children do you think your husband/partner wants to have? Why?

General Questions about Family Size

1. What do you think is the best number of children a family should have? Why?
   1. How many children would be too few for a family to have?
   2. How many children would be too many for a family to have?
2. What situations influence how many children a couple has? How do they do this?
3. How many children do other families in your area/your friends have? Do you think they have too few? Do you think they have too many?
4. What other people influence how many children a couple has? How do they do this?

Personal Questions about Pregnancy/Birth Spacing

1. Tell me about your plans for pregnancy/birth spacing plans.
   1. (If they wish to get pregnant again) When do you wish to become pregnant again? Why? What influences this wish?
2. How soon do you think your husband/partner want you to become pregnant? Why?
3. How soon do you think your friends/relatives want you to become pregnant? Why?

General Questions about Pregnancy/Birth Spacing

*Now I’d like to ask you questions about pregnancy and birth spacing. Picture another woman around your age in your community.*

1. Tell me about the ideal time period for a woman to wait after having a baby before getting pregnant again.
   1. *If need a more specific prompt*: How long should a woman wait between having a baby and getting pregnant with the next baby?
   2. What if the baby was not alive: either not alive when it was born (stillborn) or died after it was born: would the time she should wait between pregnancies change?
      - 1. If yes, how should this time change? (*ie shorter or longer, how much shorter or longer)*
        2. If yes, why should this time change?
   3. What time period between having a baby and getting pregnant with the next baby is too short? Why? What if the first baby was not alive?
   4. What time period between having a baby and getting pregnant with the next baby is too long? Why? What if the first baby was not alive?
2. Tell me what you think your husband/partner would say is the best time to wait between having one baby and getting pregnant with the next baby.
   1. What do you think your husband/partner would say is too short or too long after having a baby that is alive? Why?
   2. What about if the baby was not alive? Why?
3. Tell me what you think your friends and relatives would say is the best time to wait between having one baby and getting pregnant with the next baby.
   1. What do you think your friends and relatives would say is too short or too long after having a baby that is alive? Why?
   2. What about if the baby was not alive? Why?
4. Tell me about what would influence a woman to try to become pregnant less than six months after having a baby that doesn’t live.
   1. What are the advantages or positive things that could happen to the mother or next baby if she waited more than 18 months of a woman getting pregnant less than six months after having a baby that doesn’t live?
   2. What are the disadvantages or negative things that could happen to the mother or next baby of a woman getting pregnant less than six months after having a baby that doesn’t live?
5. Tell me about what would influence a woman to wait more than 18 months to try to become pregnant after having a baby that doesn’t live.
   1. What are the advantages or positive things that could happen to the mother or next baby if she waited more than 18 months to get pregnant after having a baby that didn’t live?
   2. What are the disadvantages or negative things that could happen to the mother or next baby of a woman getting pregnant more than 18 months after having a baby that doesn’t live?

Family planning experiences and beliefs

Now I would like to talk about family planning-the various ways or methods that a couple can use to delay or avoid a pregnancy.

1. What family planning methods you have heard about?
   1. For methods not mentioned spontaneously, ask: Have you heard of (method): *Examples: female sterilization, male sterilization, pill, IUCD, Injectables, implants, male condom, female condom, rhythm/periodic abstinence, withdrawal, emergency contraception.*
2. Have you ever used a family planning method? Which one(s)? Why did you use this/these?
   1. If yes: what did you like about the family planning methods you have used?
   2. If yes: what did you not like about the family planning methods you have used?
3. Are you currently using a family planning method now?
   1. If so, which one? When did you start using that method? Why did you choose to use that method? What do you like about the method you are using? What do you not like about the method you are using? Does your husband/partner know you are using this method? How was your husband/partner involved in deciding to use this method? Are your friends/relatives aware of your decision to use a family planning method? If so, what do they think about this decision?
   2. If not, why not? Do you want to use a family planning method now? If so, what has prevented you from using one? Is your husband/partner aware of your decision not to use a family planning method? How is your husband/partner involved in this decision? Are your friends/relatives aware of your decision not to use a family planning method? If so, what do they think about this decision?
4. Which family planning methods are best for women who have just had a baby? Which methods are not good for women who have just had a baby?
   1. If a woman’s baby is not living should she use different methods than women who have a living baby?

Feasibility and acceptability of birth spacing promotion for women who have experienced a stillbirth or neonatal death

1. What would encourage more women to use family planning after having a baby that isn’t living? Why?
   1. What would discourage women from using family planning after having a baby that isn’t living? Why?
2. When would be the best time (*ie how many weeks or months after giving birth)* for women to discuss family planning after having a baby that isn’t living? Why?
3. Who should discuss family planning with women who have a baby that isn’t living?
   1. Should HSAs discuss this? Should nurses discuss this? Should clinicians discuss this? Why or why not?
4. Where should family planning be discussed for women who have a baby that doesn’t live?
   1. Would any of these places be acceptable: home, family planning clinics, a postnatal clinic?
   2. Should the woman go back to the place where she delivered? Why or why not?
   3. Would it be useful to have a special medical clinic and provider for women who have a baby that doesn’t live? Why or why not? What would you hope that this would accomplish? What would you hope that it would not do?

This marks the end of our interview. Thank you for taking the time to answer these questions. Thank You! ! ! ! !

Finish time……………….
